# Healthcare Digital Twins Across Scales: A Narrative Review and Five-Level Conceptual Framework

**DOI:** 10.3390/healthcare14162445

**Published:** 2026-08-07

**Authors:** Leonard Azamfirei, Dorin Bica, Andrei Calin Dragomir, Emoke Almasy

**Affiliations:** 1 Department of Anaesthesiology and Intensive Care Medicine, George Emil Palade University of Medicine, Pharmacy, Science, and Technology of Targu Mures, Gheorghe Marinescu 38, 540139 Targu Mures, Mures County, Romania; leonard.azamfirei@umfst.ro (L.A.); emoke.almasy@umfst.ro (E.A.); 2 Department of Electrical Engineering and Information Technology, George Emil Palade University of Medicine, Pharmacy, Science, and Technology of Targu Mures, Gheorghe Marinescu 38, 540139 Targu Mures, Mures County, Romania; 3 Doctoral School of Medicine and Pharmacy, George Emil Palade University of Medicine, Pharmacy, Science, and Technology of Targu Mures, Gheorghe Marinescu 38, 540139 Targu Mures, Mures County, Romania; andrei.dragomir@umfst.ro

**Keywords:** digital twin, narrative review, precision medicine, health system intelligence, validation, interoperability, governance, clinical decision support, healthcare simulation, learning health system, digital health

## Abstract

**Background/Objectives:** Healthcare digital twins are being developed at scales ranging from individual organs to regional health systems. However, the literature at these different scales has largely evolved independently. This narrative review examines how the concept changes as the represented object grows, and proposes a five-level framework: physiological, patient, care-delivery, hospital and health-system twins proposed by the authors as an analytical framework. **Methods:** Web of Science, Scopus, PubMed and Google Scholar were searched between June and July 2026 for English-language records published from January 2022 onwards. Sources were eligible if they described a virtual representation of an identified physical counterpart in healthcare, updated from that counterpart’s own data and used to produce decision-relevant predictions. Editorials, abstract-only records, static models and work outside healthcare were excluded. The included literature was examined to identify recurring patterns in the representation and application of healthcare digital twins. These patterns informed the development of the authors’ proposed five-level conceptual framework. As no formal quality appraisal was undertaken, conclusions regarding evidence maturity and implementation should be interpreted cautiously. **Results:** Forty-four publications form the evidence base: 10 original studies, 26 reviews and 8 conceptual or consensus papers. Patient-specific applications were described primarily at the physiological and patient levels, particularly in cardiology and oncology. External validation remained uncommon, and prospective evaluation was rare across all five levels. Care-delivery twins were reported mainly in critical care, emergency and perioperative settings, whereas hospital and health-system applications were largely limited to prototypes and simulations. **Conclusions:** This review suggests that the challenges associated with healthcare digital twins evolve as applications move from physiological models to health-system settings. Beyond technical complexity, interoperability, organisational, governance and equity considerations become increasingly important. Prospective evidence of patient benefit, operational effectiveness and system-level impact remains limited across all five levels.

## 1. Introduction

Modern health systems generate more data than they use. Electronic records, imaging archives, genomic panels, wearable sensors and hospital operating systems produce information continuously, yet much of it remains stored rather than translated into timely clinical or operational decision support [1]. Existing infrastructure was built primarily to record and retrieve data, with limited capacity to integrate predictive models and simulations. Consequently, more data has not consistently translated into better decisions [1].

A second difficulty arises from both biological and organizational complexity. Diseases arise from interactions among molecular, cellular and environmental factors that differ between patients and shift over time, so findings based on population averages do not always transfer to the individual in front of the clinician [2]. Hospitals face a structurally similar problem. Bed occupancy, staffing and patient flow depend on many interacting constraints, and dashboards often reveal pressure only once it has built [3]. At both levels, a representation that links data, mechanism and simulation before decisions are made is still lacking [1,3].

One approach that has attracted growing attention is the digital twin [4]. The concept originated in engineering and aerospace, where a virtual model remains aligned with its physical counterpart through data exchange, allowing engineers to monitor, simulate and optimise the object without interrupting its operation [5]. In healthcare, however, the term should be used carefully. A digital twin is not merely a 3D model, a risk score, or a one-time simulation. This review uses an operational definition informed by the United States National Academies’ framework (2024): a virtual representation linked to a specific physical counterpart through bidirectional data exchange, updated over time, and capable of generating predictions that inform decision making [4]. We selected this definition because it provides clear criteria for identifying a digital twin and was developed with contributions from engineering, biomedical and computational disciplines. It is not universally accepted, and alternative definitions differ in how they address personalisation, data updating, bidirectional coupling, and automated actuation. As a result, some studies included in this review would not meet all of these criteria [6]. Bidirectional coupling distinguishes a digital twin from digital shadows, virtual cohorts and standalone predictive models, but these terms remain inconsistently used in the healthcare literature [6].

In healthcare, early digital twin applications focused on representing individual patients [7]. In most cases, a computational model of a patient’s biology is built, interventions on that representation are tested in silico, and the results are used to inform treatment selection [8]. The focus has since expanded beyond the individual patient. Processes and institutions can also be modelled as twinned systems, and recent studies have extended this approach to clinical pathways, departments and hospitals, integrating clinical, spatial and operational hospital data into a single layered model [1,9]. More recently, the same concept has reached populations, regional health systems and learning health systems, where organisations use their own data in order to improve clinical practice [2,3,10].

That outward movement should not be seen as a simple extension of the same object. A hospital is not simply a larger patient. Healthcare organisations are adaptive sociotechnical systems shaped by multiple stakeholders, operational constraints and governance requirements [11,12]. Accordingly, we propose a framework in which each level is treated as conceptually distinct, reflecting the different questions, decisions and constraints that arise at each scale. Fields that adopted digital twins earlier report the same pattern. A review from the construction sector found that structured education and training can help learners apply digital twin concepts in practice and develop relevant workforce skills [13]. Although healthcare is different, staff preparation is also likely to be important when these systems are introduced into practice [13]. Healthcare inherits both the concept and that dependence on capability as much as on technology.

The expansion of digital twins has produced less consistency about the concept [6]. One review reported that only 18 of 149 studies describing a digital twin met the full criteria of a personalised, dynamically updated and predictive twin. Digital shadows, generic digital models and virtual patient cohorts each accounted for around a tenth of the total, and only two studies mentioned verification, validation and uncertainty quantification [6]. A meta-review grouped applications into personalised medicine, operational efficiency and medical research, and identified data quality, ethical governance and socioeconomic disparities as recurring obstacles [12]. Bibliometric work has documented the growth of the field [14].

What these reviews leave unresolved is the transition across scales. They organise the literature by organ, disease, technology or use case, with cardiac, oncological and hospital twins discussed separately rather than within a common cross-scale framework. Although some reviews discuss the maturity of different application areas, they do not compare them using a consistent framework. They also discuss validation, interoperability and governance as general challenges, even though the requirements differ across levels. The question asked of a cardiac model is not the same as the question asked of a regional capacity model, and neither the evaluation criteria nor the responsible stakeholders are the same [1,12].

We use the term health system intelligence to describe the capacity of a health service to sense its own state, simulate the consequences of alternative decisions, and act on the results in a continuous loop across patient, process, institution and population. Throughout this review, the term is used as a proposed organising concept rather than an established construct. It overlaps with, without being identical to, several neighbouring ideas, and the boundaries drawn between them here reflect our own reading rather than settled definitions. Learning health systems are concerned principally with returning routine care data to practice improvement, and that loop need not include a coupled simulation layer [2,10]. Precision public health stratifies risk across populations, which does not by itself require a maintained live representation of the service delivering care [15]. Systems medicine addresses biological complexity rather than organisational behaviour. Existing human digital twin architectures describe the technical components needed for sensing, communication, computation and decision support, but not how those capabilities would together constitute health system intelligence [16]. What we intend by the term is the combination: coupled representation and simulation, applied at more than one organisational scale, feeding decisions whose consequences are then observed.

Precision medicine is broader than the use of a digital twin for an individual patient. It also includes biomarker-based stratification, molecular characterisation, prognosis, prevention and treatment selection informed by molecular, clinical and population-level evidence. Digital twins represent one way of bringing these elements together rather than defining precision medicine itself [7,17]. The two concepts overlap, but their differences go beyond scale.

This review addresses four main questions. First, what operational definition of a healthcare digital twin is used in the current literature, and how consistently is it applied? Second, can that literature be organised as a set of representational scales, and what defines each level? Third, how does the reported maturity of the evidence differ between levels, and on what basis can that difference be judged in the absence of formal quality appraisal? Fourth, how do the requirements for validation, interoperability and governance change as the represented object grows?

A distinction runs through all four. Predicting what will happen is not the same as estimating what would happen under an alternative action. A model developed to forecast deterioration cannot automatically be assumed to provide reliable estimates of alternative interventions. Whether a model is appropriate depends on its intended purpose and the evidence supporting its validation for that application [4,17]. Wherever a twin is described as supporting treatment selection or policy simulation, the causal or counterfactual assumptions that would make such a claim valid are the substantive question, and they are seldom stated [17,18].

The clinical case for these systems is usually made in terms of anticipation. The risks deserve equal prominence. Erroneous recommendations, automation bias, model drift, feedback instability once recommendations begin to alter the process generating the data, optimisation that is efficient in aggregate but inequitable in distribution, cybersecurity compromise of a bidirectionally connected system, and inappropriate allocation of scarce resources are all plausible failure modes. Several become more consequential as scale increases [11,17,19].

This review begins by asking how a healthcare digital twin should be defined and distinguished from adjacent constructs. It then considers whether the applications described across healthcare form a single continuum and where the boundaries between scales lie. Finally, it examines the validation, interoperability and governance requirements that must be met before these systems can be used in real populations.

## 2. Materials and Methods

### 2.1. Design and Rationale

This is a structured narrative review: the sources searched, the eligibility rules and the selection process are stated explicitly, but no claim is made to exhaustive retrieval and no results are pooled [20,21]. A scoping or systematic mapping review would have produced a more reproducible inventory. The central difficulty in this field is definitional rather than enumerative: the same label is applied to objects ranging from an organ model to a proposed regional planning infrastructure, so a count of records tagged ‘digital twin’ would measure labelling practice more than capability [6]. Our objective was conceptual, namely to establish whether a cross-scale reading of this literature is coherent. We therefore chose a narrative design that retains as much procedural transparency as that objective allows [22].

**Figure 1:**
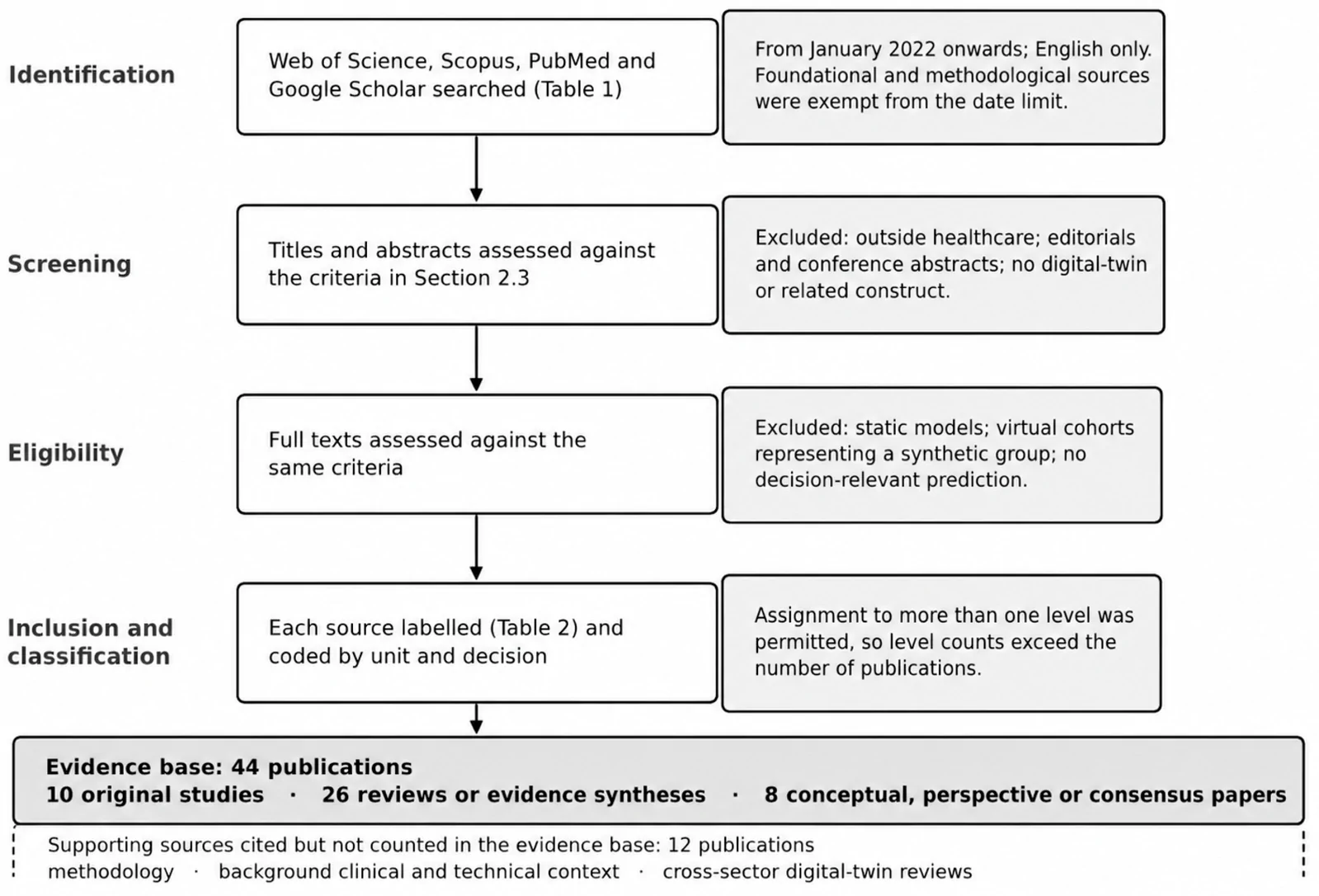
Source selection and classification, showing the criteria applied at each stage and the composition of the resulting evidence base. This is not a PRISMA flow diagram: record-level yields at each stage were not retained during the conduct of the review and are therefore not reported. Supporting sources shown in the lower box were cited for methodological guidance, background clinical or technical context, or cross-sector comparison, and are not part of the evidence base.

Reporting follows the published methodological guidance for narrative reviews [20,21,22]. The PRISMA 2020 statement, which applies to systematic, scoping and umbrella reviews, was consulted for the structure in which source selection is described, and Figure 1 follows that structure without claiming PRISMA compliance [23]. Transparency is provided through the sources searched and search strings (Table 1), the eligibility criteria (Section 2.3), the selection process (Figure 1), the classification scheme (Table 2), the characteristics of the included publication, and the assignment of sources to levels.

No protocol was registered and no prespecified analytical plan was lodged in advance. Inclusion boundaries and the classification scheme were therefore developed during the review, and not before it, and may have been shaped by the sources encountered. We report this explicitly because it bears on how the framework in Section 3.2 should be read.

### 2.2. Search Strategy

Four sources were searched: Web of Science Core Collection, Scopus, PubMed and Google Scholar. Table 1 gives, for each source, the platform, the complete search string including Boolean operators, field restrictions, controlled vocabulary and truncation, and the filters applied. The searches were run between June and July 2026.

The search window for the applied literature was 1 January 2022 to the final search date. That window was chosen because the first consolidated healthcare-specific reviews and definitional statements appeared from 2022 onwards, and because implementation reports in this field describe infrastructure that is superseded quickly [14,24]. The restriction was not applied to foundational, definitional and methodological sources, which were retained irrespective of publication year and identified by hand-searching the reference lists of included reviews. This is why the reference list contains material published before 2022 [5,22]. No forward citation searching, expert consultation, grey-literature searching or trial-registry searching was undertaken, and no unpublished or vendor-held implementation reports were sought.

Google Scholar ranks results by relevance and may personalise them, so it is not reproducible in the same way as the three indexed databases. It was used only as a supplementary check to identify additional relevant publications. No source was included solely on the basis of the Google Scholar search. The search strategies for the three indexed databases are reported in full in Table 1 to support reproducibility.

### 2.3. Eligibility Criteria

A source was eligible for the evidence base if it described, in a healthcare setting, a virtual representation that met the first criterion and addressed the remaining two: (i) representation of an identified physical counterpart: a specific organ, patient, unit, institution or population, rather than a generic or averaged entity; (ii) updating of that representation from data generated by the same counterpart, at an interval appropriate to the decision it was intended to support; and (iii) generation of predictions directed at a clinical or operational decision.

Partial compliance was permitted. Requiring all three criteria would have excluded much of the available literature and prevented comparison across scales. Sources that did not satisfy all three were retained but labelled, using the scheme in Table 2, as proto-twins, digital shadows, dynamic simulations, predictive models or conceptual architectures, not as full digital twins [6]. Sources that used the label ‘digital twin’ while describing a static three-dimensional model, a one-off simulation with no counterpart-specific updating, or a virtual cohort representing a synthetic group, rather than an identifiable individual, unit or organisation, were recorded as boundary cases and are reported as such, and not counted as digital-twin evidence.

Exclusions were: publications outside healthcare; editorials, commentaries, conference abstracts and abstract-only records; and publications not available in English. The English-language restriction is stated here as an eligibility criterion, and not only in the limitations, because it is a source of selection bias, not an incidental feature.

Eleven sources cited in this article fall outside these rules by design. Methodological references on the conduct of narrative reviews, background clinical and technical references cited for context, and cross-sector digital-twin reviews from other industries cited for comparison are identified as supporting sources [13,20,22,25]. They are not counted in the evidence base and contribute to no maturity statement. The 44 publications that constitute the evidence base are listed later in this article; any reference cited in this article but absent from that list is a supporting source. This distinction makes clear which references contributed to the evidence base and which were cited only for context.

### 2.4. Screening and Selection

Records from the four sources were combined and duplicates identified by matching on title, first author, journal and digital object identifier, then removed before screening. Titles and abstracts were screened against the eligibility criteria, and records retained at that stage were assessed in full text. The literature search and study selection were performed by a single author. Records that were uncertain or borderline against the criteria, and the assignment of sources to levels, were discussed among the authors. Screening was therefore not conducted in independent duplicate, and no inter-rater agreement statistic or formal consensus procedure was used. Each publication was assigned to the level that best represented its primary application. The classifications should therefore be interpreted with this limitation in mind: the term ‘digital twin’ is applied inconsistently across the literature, so classification rests on judgement, and a single screener carries a higher risk of misclassification than independent double screening [6]. Figure 1 summarises the sequence of selection and classification decisions and the criteria applied at each stage.

### 2.5. Data Extraction and Classification

The following characteristics were recorded for each included source: publication type; clinical or operational domain; the physical counterpart represented; the level or levels to which the source was assigned; data sources; model family; updating interval; directionality of coupling; whether the system could inform or execute an intervention; validation design; deployment status; and reported outcomes. The fields that could be populated consistently across the whole corpus are reported later in this article. The remaining fields were recorded where a source reported them, but are not tabulated per source: reporting was too incomplete for a complete table, and a table with a majority of cells marked as not reported would convey the gaps in the primary literature, not the characteristics of the corpus. Where those fields inform a statement in Section 3, the statement is attributed to the source that reports them. Absence of reporting was recorded as such and was not treated as absence of the property.

The included literature was then reviewed to identify recurring patterns in the representation and application of healthcare digital twins, principally the unit that each source represented and the decision the representation was intended to support. These recurring patterns formed the basis of the five-level conceptual framework proposed by the authors, which is intended to facilitate the classification of healthcare digital twins. No formal qualitative coding, thematic analysis or comparable structured qualitative methodology was applied. As this is a narrative review, the framework reflects the authors’ interpretation of the included literature rather than the result of a formal analytical procedure. No pre-existing taxonomy was imposed, although the resulting levels were compared afterwards against published classifications, as set out in Section 3.2.

The unit of classification is the object represented, not the setting in which the work was carried out. A model of one patient’s physiology in an intensive care unit is a patient twin. A model of the same unit’s bed and staffing flow is a care-delivery twin, even though both are commonly described as ‘ICU digital twins’. Where a source represented more than one unit (for example, an architecture spanning ward flow and institutional capacity), it was assigned to more than one level. Counts by level therefore exceed the number of included publications. Where a source combined levels without distinguishing them, it was assigned to the higher level and the ambiguity was recorded.

Reviews and the primary studies they summarise are both present in the evidence base. To avoid weighting the same finding twice, a primary study encountered only inside an included review was not added as a separate record, and statements drawn from a review are attributed to that review, with the underlying evidence identified as secondary where this matters to the argument.

### 2.6. Labelling of Status and Maturity

Maturity is used throughout this article, and it is not a single property. Reporting it as one conceals the fact that a field can be technically sophisticated and simultaneously unvalidated and unimplemented. We therefore separate three dimensions and report them independently: technical development, validation, and implementation [17]. Table 2 defines the labels used within each dimension, together with the labels used to record whether a described system meets the definitional criteria in Section 2.3.

No formal quality appraisal, risk-of-bias assessment or critical-appraisal instrument was applied to any included publication. This is the most important constraint on everything that follows: a label in Table 2 records what a source reported and demonstrated, not how well the underlying work was conducted. We did not apply an instrument for two reasons. No common instrument exists for a corpus containing mechanistic modelling studies, discrete-event simulations, scoping reviews and proposed architectures, and appraising only the minority of comparative studies would have produced a partial assessment liable to be read as though it covered the whole. All maturity statements below are therefore provisional author judgements and are presented as such rather than as graded evidence.

### 2.7. Synthesis

Synthesis was narrative and organised by claim, not by source. No meta-analysis was performed. Quantitative pooling was precluded by heterogeneity at three levels: the objects represented differ in kind; the models belong to different families, from mechanistic partial-differential-equation systems to discrete-event simulation; and reported outcomes range from model error metrics through bed occupancy to epidemic case counts, with no common effect measure.

Two restricted readings of the corpus were carried out as informal checks on the descriptive findings, and not as statistical sensitivity analyses. The first considered only the original empirical studies. The second was intended to consider only systems satisfying all three definitional criteria, but too many publications reported insufficient detail to establish whether the criteria were met, and it could not be completed as intended. Both are reported in Section 3.7.

Where sources disagreed on definitions, on whether a given system qualifies as a twin, or on how ready the field is for clinical use, the disagreement is reported and attributed rather than resolved by preferring one account [6,26,27].

## 3. Evidence Synthesis

### 3.1. Conceptual Foundations: Defining the Healthcare Digital Twin

The concept originated in engineering and aerospace, where a virtual representation of a physical object is updated by sensor data, allowing its behaviour to be simulated and optimised [5].

Three properties characterise a healthcare digital twin under the definition adopted here: representation of an identified counterpart, coupling to that counterpart through data flowing in both directions, and predictive capacity directed at a decision [4,28]. None is sufficient alone. A model may be anatomically detailed, or frequently updated, or predictive, without being a twin [4].

A faithful virtual representation cannot be assessed directly. In this review, we assess fidelity through calibration, mechanistic adequacy for the intended application, predictive performance, agreement between the modelled and observed state, and resilience to incomplete or degraded inputs [17]. Continuous updating is likewise better treated as a scale than as a switch. What matters, on our reading, is whether the update interval is short relative to the decision horizon. The horizon differs between intensive care, chronic disease management and capacity planning. The same system may therefore be adequately synchronised for one purpose and inadequate for another, so we treat ‘continuously updated’ as meaningful only once the decision it serves is specified.

This distinguishes the digital twin from several related concepts, all of which are defined in Table 2. Conceptually, a static three-dimensional model represents structure without a living data link, whereas a one-time simulation reproduces behaviour but it is not updated continuously. A virtual cohort represents a group of virtual individuals rather than a specific patient. Similarly, a predictive algorithm may support decision making without closing the loop with the entity it represents [6,8]. The digital shadow is the closest concept, and it differs in one respect only: it may be updated repeatedly from its counterpart, in some implementations continuously, but nothing runs back from the model to the entity it represents [6,8]. The term is currently used for systems ranging from organ models to proposed regional planning infrastructures [1,11,14].

Bidirectional coupling and a closed decision loop are related but not equivalent, and the two are frequently conflated [6]. Coupling is a property of the data path alone: observations travel from the counterpart to the model, and something travels back towards the counterpart’s environment. A closed loop is a property of the whole arrangement. It requires in addition that a decision is taken, that an action follows, and that the consequence of that action returns as data. A system can therefore be bidirectionally coupled while still leaving the loop open if the returned information is observed but not acted upon.

In this review, we distinguish five stages between the two, and these differ in what they require and in who is answerable for them. Data exchange is the movement of observations into the model and of model state back out [4]. It is primarily a technical property. Recommendation generation turns model output into something a person can act on, which involves choices about thresholds, ranking, timing and presentation that the model itself does not contain. Human decision making is the point at which a clinician or manager judges whether the recommendation fits the case in front of them. Professional accountability is centred here, as is the possibility that the recommendation is rejected, modified, delayed or applied incompletely [4]. Physical intervention is the act that changes the counterpart, and it may differ from what was decided, because a resource was unavailable or because the situation moved on while the decision was being made. Automated actuation removes the third stage altogether: the system acts on its counterpart with no human judgement between output and effect. In engineering applications this is common, and it is what closed the loop in the original sense of the term [4,5]. In healthcare literature reviewed here, it is the exception. None of the reviewed publications described a twin that actuates an intervention without a human decision in between, and the accountability consequences of removing that stage are not addressed in the literature reviewed here.

**Figure 2:**
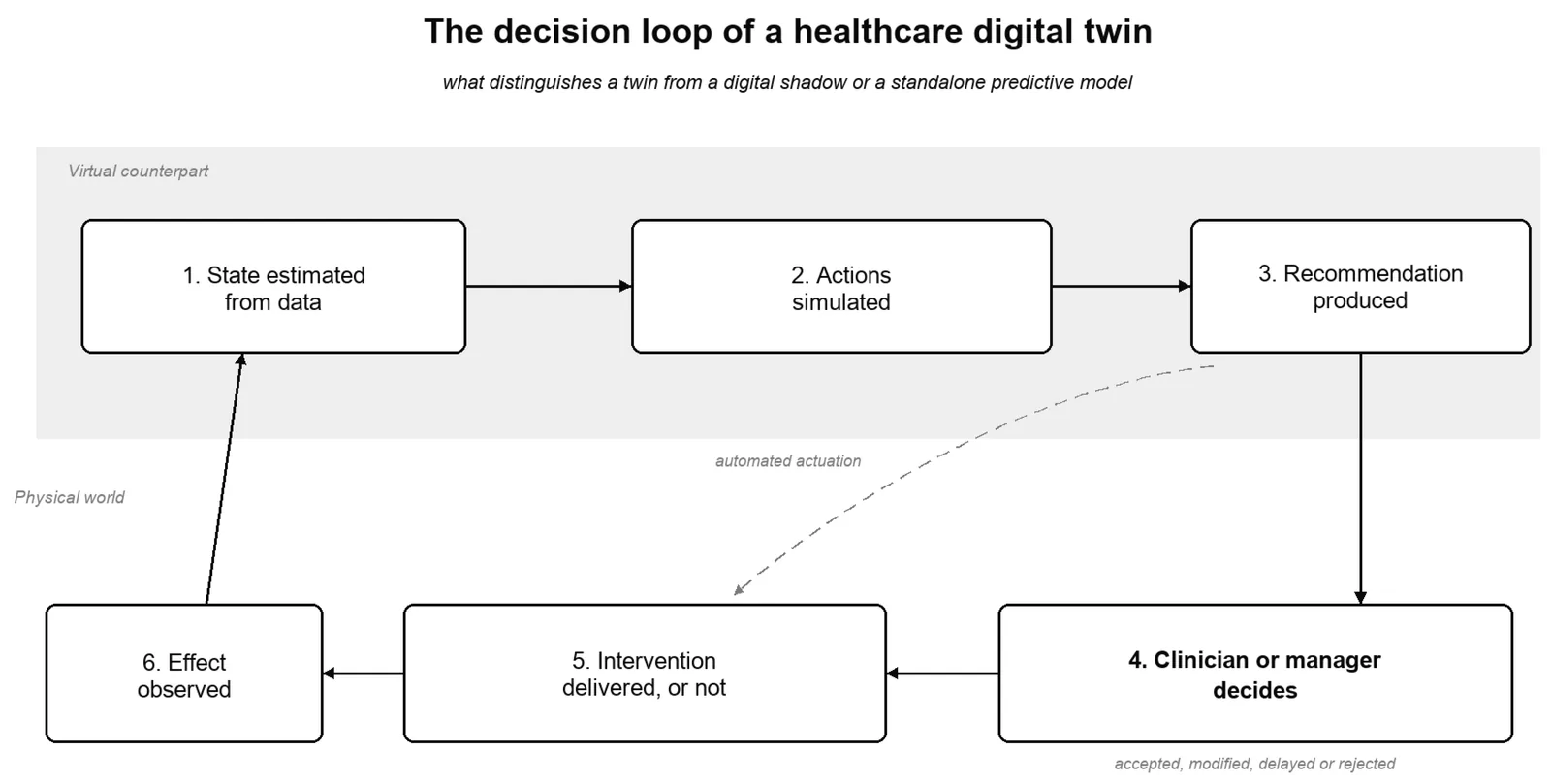
The decision loop of a healthcare digital twin, with data exchange, recommendation generation, human decision making and physical intervention shown as separate stages. Human judgement and accountability lie between recommendation and intervention, and the recommendation may be accepted, modified, delayed, rejected or applied incompletely. The dashed path marks automated actuation, in which the system acts on its counterpart with no human decision in between. None of the reviewed studies described such a system.

Figure 2 separates these five stages and marks the actuation path as an exception, rather than the norm. The distinction matters for classification as much as for governance: a system that exchanges data in both directions satisfies the coupling criterion of Section 2.3, but it closes the loop only when a decision and an intervention follow and are observed. Several of the systems described in the sources reviewed here satisfy the first condition but not the second. Representing the loop as deterministic would not reflect the role of clinical and organisational judgement in healthcare digital twins [11].

Accordingly, the statement that only a twin closes the loop applies to the operational definition adopted in this review, rather than universally. Frameworks that accept periodic updating or regard a human-mediated feedback path as sufficient would classify several of the systems that we describe as proto-twins as full digital twins [6,26]. Our aim is simply to apply one operational framework consistently throughout this review, while acknowledging that other classification approaches exist.

### 3.2. A Proposed Five-Level Framework

Based on the reviewed literature, we propose a five-level conceptual framework for classifying healthcare digital twins. If a patient can be represented as a dynamically updated counterpart, it does not follow that the care process, hospital and health system around that patient can be represented by direct extension. Each level below is therefore proposed on its own conceptual basis.

**Figure 3:**
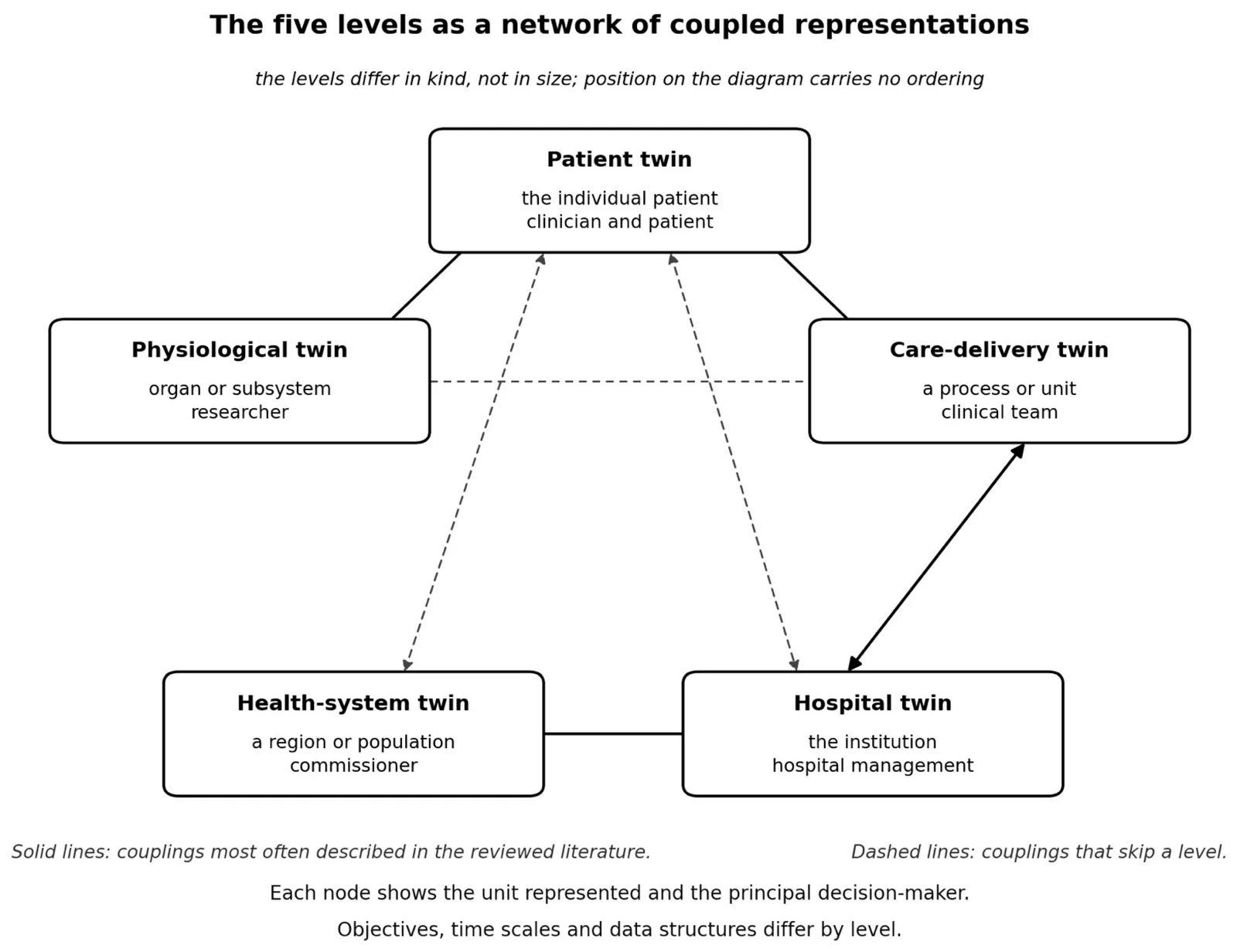
The five levels are shown as a network, and not a sequence. Position on the diagram does not indicate an ordering, and no level precedes, contains or aggregates into another. Solid lines mark the couplings most commonly described in the reviewed literature and dashed lines mark couplings that skip a level, such as patient-level data feeding institutional capacity models directly. Each node identifies the unit represented and the principal decision-maker. Objectives, time scales, data structures and the remaining differences between levels are summarised in Table 3. The levels differ in kind, not in degree, and no ordering of maturity, evidence strength or importance is implied.

The physiological twin represents an organ, tissue or biological subsystem and reaches down to molecular mechanism. It forms a distinct level because its validation question is mechanistic and because its counterpart may be a structure, not a person [29,30]. The patient twin centres on the individual, and is the level at which computational precision medicine operates. It is distinct because it must integrate heterogeneous data about one named person, and because consent, privacy and clinical accountability attach to that person [7,8]. The care-delivery twin represents a clinical process or unit. It differs from the previous levels because its object is a workflow with queues, capacity and staff, and because its outcomes are timing, safety and resource use rather than physiology [31]. The hospital twin represents the institution as an operating system, with cross-departmental interactions and a management decision-maker [9]. The health-system twin focuses on a regional or population network in which several organisations with separate governance and competing objectives must be reconciled [2,3]. Table 3 summarises the five levels, and Figure 3 shows them as a network of coupled representations.

The boundary between physiological and patient twins is not sharp. An organ model personalised to one individual satisfies both descriptions. We resolve this by applying a consistent classification rule: a source is assigned to the physiological level when the object of the model is the organ or subsystem and the question concerns mechanism, and to the patient level when the object is the person and the question concerns that person’s management. Sources meeting both criteria are assigned to both. The same rule also applies to the boundary between care-delivery and hospital twins. In such cases, a pathway spanning the emergency department, intensive care unit and operating theatre is classified by the unit whose behaviour the model is used to change, instead of the departments it traverses.

Within the operational framework adopted in this review, device twins are classified according to the entity represented. When a device is modelled together with an identified patient, it falls within the physiological or patient level. By contrast, where the counterpart is a product or device family rather than an identified user, it falls outside the framework. Therapy twins are placed at the patient level because the treatment is evaluated against a representation of an identified patient. The same principle places pharmaceutical-development twins outside the framework: although they rely on virtual populations standing in for trial cohorts, they do not represent an identified physical counterpart. This is the same distinction that excludes virtual cohorts in Section 2.3. Building-infrastructure twins require a similar distinction. They are classified at the hospital level only when the building model is coupled to the clinical and operational activity of the institution. A model concerned solely with ventilation or energy use represents a building, rather than the hospital as a healthcare organisation. Public-health models are treated in the same way. They fall outside the framework unless they remain linked to an identified healthcare service or population registry. For this reason, the pandemic models discussed in Section 3.6 are classified as simulations within the operational definition adopted in this review [32,33]. Two of these five therefore fall wholly or partly outside the framework. We consider this preferable to broadening the taxonomy until it encompasses everything but distinguishes nothing.

Aggregation across levels is not straightforward and should not be assumed. Summing patient twins does not produce a hospital twin, because organisational behaviour depends on capacity constraints, clinician decisions, policy and interactions between patients that have no representation in any individual model [12,31]. What the levels share is a structure, not a construction path. The relationship between them is reciprocal rather than one-way: unit-level models draw on patient-level data, and unit-level outputs change the conditions under which individual care is delivered [3]. The framework proposed here is intended to allow the literature to be read along an individual-to-collective axis, whereas on our reading existing reviews mostly keep these levels apart [14].

Table 4 sets the proposed framework against the classifications already available. Previous schemes have grouped applications by purpose, by clinical domain, by technical architecture, by definitional compliance, or by decision type [1,6,12,14,16,34]. These are useful and we do not replace them. What the five-level scheme adds is a single axis, the unit of representation, along which validation questions, decision-makers and governance requirements can be compared, and against which a source can be located even when its clinical domain differs from every other source in the corpus.

### 3.3. Physiological and Patient Twins

Concrete, patient-specific applications are represented more frequently at the physiological and patient levels than elsewhere in this corpus, and they are concentrated in cardiology and oncology [7]. That is a statement about where applications are described. It is not a ranking of validation or implementation maturity, and on those two dimensions the picture at these levels is no stronger than at any other.

At the physiological level, single-cell frameworks use molecular data to identify disease genes and therapeutic targets [29]. Cardiac models increasingly combine mechanistic simulation and statistical learning to predict disease evolution and treatment response [30,35]. Recent studies explore how generative artificial intelligence could extend these models [36]. Another area where digital twins are being developed is immuno-oncology [37,38,39].

A mechanistic model is not a digital twin merely because it is mechanistic. It qualifies at this level only if it is linked to an identified counterpart and updated from data generated by that counterpart. Several of the physiological examples above are personalised at initialisation and then run forward without further counterpart-specific updating, which places them closer to the proto-twin label of Table 2 than to a fully coupled twin [29,37]. Biological plausibility is also not equivalent to validity: a model may incorporate correct mechanisms and remain poorly identifiable from the data available, inadequately calibrated for the population in which it is used, or non-transportable to another setting [17].

At the patient level, the aim is a representation of one individual’s biology, updated from that individual’s data and used to compare candidate interventions before one is selected [8]. This is the level closest to computational precision medicine [7,8]. Reviews report early clinical applications that are better kept in separate categories than combined into one list: disease areas, principally oncology and cardiology; device- and organ-support applications; and operational uses that belong at the care-delivery level, not here [3,12,26]. Whether the approach is ready to influence clinical evidence generation or routine practice remains contested, and that disagreement is substantive, not terminological [27,40]. Building a dependable twin from data that are incomplete, irregularly sampled and not collected for modelling remains a recurring obstacle [18,28].

The data used to construct patient twins are dominated by biological, imaging, genomic and device sources [16,41]. Behaviour, treatment adherence, patient preference, social circumstance and environmental exposure are largely absent, and the omission is not a matter of completeness alone. Each of these operates on the twin in a different way, and each breaks a different assumption.

Adherence sits between the recommendation and its effect. A model that simulates a drug regimen simulates the regimen as prescribed, not as taken, and where adherence is partial the predicted trajectory describes a patient who does not exist. Preference determines whether the option the model ranks first is the option the patient would choose at all. A twin that optimises survival for a patient who is optimising for independence or for time at home has answered a question nobody asked. Social circumstance (housing, income, caring responsibilities, transport, food security, health literacy) shapes both what is measurable and what is achievable, and it is generally recorded in health records sparsely, inconsistently and with systematic differences between groups. Environmental exposure operates on the physiology the model represents while sitting almost entirely outside the data that populate it. And access to the devices that generate continuous data is itself socially patterned, so the patients whose twins would be best supplied with data are not a random sample of patients [19,42].

The consequence is not simply that predictions are less accurate. It is that they may be unevenly accurate in a direction that tracks existing disadvantage, and that a twin built on biological data alone will attribute to biology variation that arises from circumstance. The review of digital twins for people living with disability highlights a broader view of what a healthcare digital twin can be. Alongside physiological modelling, many applications centre on rehabilitation, assistive technologies, and continuous monitoring, reflecting the wider context in which care is delivered [43]. Generalisability is a further constraint: a patient twin developed in one population may not transfer to another [17,18].

### 3.4. Care-Delivery Twins

Care-delivery twins represent the clinical process around the patient, and not the patient alone [1]. Two model types are described under this heading and they are not interchangeable. The first represents an individual patient trajectory within a care process. The second represents workflow, capacity and resource allocation for a unit. Their data, their validation requirements and their failure modes differ, and we treat them separately.

Critical care is where trajectory-level work is concentrated, because decisions are frequent, data volume is high and a patient’s state can change quickly [44]. Models have been built to anticipate how critically ill patients are likely to progress and to compare management strategies before they are applied. In haemorrhagic shock, mathematical models have been used to suggest individualised resuscitation strategies [45]. In acute stroke, expert-consensus rules have been developed for a digital-twin model of care in a neurocritical care unit [46]. In sepsis, dynamic trajectories of cardiorespiratory failure have been used to inform intensive care unit twins [47]. Prediction remains difficult, partly because critical illness involves interacting inflammatory, coagulation-related and endocrine changes across several organ systems [25].

Unit-level models represent something else. Multidisciplinary models of critical care delivery describe patient flow, bed allocation, staff distribution and intervention pathways, and are used to identify bottlenecks and test reallocation before it is attempted [9,31]. Layered architectures for a healthcare unit organise clinical, spatial and operational data to support ward-level decisions [9]. The same logic has been applied along the rest of the clinical process: emergency-department flow and prehospital care have been examined through emergency-care twins [48], simulation-based twins have been used to assess the organisation of response to emergency calls [49], and operating-theatre scheduling has been modelled as a multi-resource constrained allocation problem [50]. Reviews of clinical and operational decision making place much of this work in the operational category [12,34].

Most of these systems are prototypes or single-site studies. Few report integration into a live workflow, and none in this corpus reports a comparative evaluation of effect on patient outcomes. Model development is only one part of the explanation for this gap. These include interoperability with existing information systems, the need to maintain models during routine clinical use, and integration into clinical workflows, including clinician acceptance [11,12]. Model performance alone is therefore unlikely to determine clinical impact [11,17].

Using such models to allocate resources also raises questions that are not technical. If a care-delivery twin recommends which patient receives the next intensive care bed, the model encodes a priority rule, and responsibility for that rule has to be located before the system is used, not after [11].

### 3.5. Hospital Twins

At institutional level the hospital digital twin, as the term is used here, represents the hospital as an operating system, not a patient. It combines clinical, operational and infrastructural data to support decisions on patient flow, bed allocation, staffing and use of infrastructure [9]. It has been proposed that a hospital twin could function as an operational command centre, in which bed management, operating theatres, the emergency department, intensive care, the workforce, critical equipment and logistics are represented together, and in which overload or a proposed reorganisation of wards could be simulated in advance [3,9,50]. We found no published account in this corpus of such a command centre operating on a live institutional counterpart, so the capability is reported here as proposed, and not demonstrated. Emergency hospital services and patient handling have also been represented as twinned processes [51].

The potential is considerable, particularly because hospital managers could identify occupancy pressures and resource constraints earlier, which is why operational efficiency appears as a major application area, and reviews of clinical and operational decision making place hospital-level twins in that category [3,12,34]. The main limitation is that most published works still describe architectures, individual cases or prototypes rather than complete and validated systems.

It would be wrong to conclude that model construction is no longer the difficulty at this level. Model specification, data quality, identifiability, computational reliability and synchronisation between a live institution and its representation are all unresolved, and semantic harmonisation across departmental systems is a technical problem before it is an organisational one [9,52]. What is true is that a second class of difficulty is added on top: integrating heterogeneous information systems, running the model continuously rather than for a study period, and producing an interface a bed manager will use at eight in the morning [11,12].

Cost is rarely addressed in the literature, and it is not a minor omission. A hospital twin implies sustained expenditure on infrastructure, software development, validation, maintenance, cybersecurity, licensing and staff with skills that hospitals do not usually employ. No source in this corpus reports an economic evaluation set against the operational benefit obtained. Whether the investment would be justified is therefore an open question, not an optimistic one [12].

Hospital digital twins are designed to optimise specific objectives, such as patient flow, resource allocation, staffing, or operational efficiency. The choice of what to optimise matters because improving one outcome may affect others. These trade-offs are often implicit, yet they can shape the recommendations made by the model. Making the optimisation objectives, and the rationale behind them, explicit is therefore an important part of digital twin governance rather than simply a technical modelling decision [11,12].

### 3.6. Health-System Twins and Learning Health Systems

Beyond individual hospitals, digital-twin approaches have also been proposed for regional health systems and populations [3]. The intended applications are governance and planning functions: regional capacity planning, epidemic preparedness, coordination of emergency medical services and inter-hospital transfer, and allocation of public-health resources [2,3,10,49]. Simulation-based twins have been used to assess the organisation of regional emergency call response and to evaluate organisational change before implementation [49], and individual-based models were used during the COVID-19 pandemic to evaluate mass testing and local lockdowns [33].

Whether the pandemic models meet the definition used in this review is doubtful. Several were calibrated to national surveillance data and run forward as scenario simulations rather than coupled continuously to a named service, and on the criteria of Section 2.3 they are better described as simulation models than as digital twins [32,33]. The pandemic increased interest in population- and system-level twins for outbreak management, and reviews have examined their potential for managing healthcare systems [32,53]. The long-term proposal is a learning health system in which patient, care-delivery, hospital and health-system representations inform one another [2,10]. At this level the object is closer to precision public health than to bedside care [15].

Three difficulties are specific to this level. The first is representational: a health system has no determinate state in the sense that a ventilator circuit does, and no source in this corpus demonstrates a dynamically coupled model of an entire service. The second is data-governance: linking several organisations requires reconciling incompatible information systems and, before that, incompatible legal bases for sharing [2,54]. The third is normative. If such a model is used to allocate resources across populations, it embeds value judgements about equity and priority that no amount of technical accuracy will settle. Equity, access, preparedness and cost-effectiveness are frequently named as evaluation criteria but are rarely operationalised, and they conflict: a configuration maximising aggregate cost-effectiveness may reduce access in sparsely populated areas. How such trade-offs are resolved, and by whom, is a governance question, and it is not answered in the literature reviewed here [11,54].

The movement towards connected system-level twins should not be presented as inevitable. Some applications may remain appropriately local, disease-specific or operationally isolated, and the case for connecting them has to be made application by application rather than assumed from the direction of the field.

### 3.7. Comparing Maturity Across Levels

The evidence base comprises 10 original studies, 26 reviews or evidence syntheses and 8 conceptual, perspective or consensus papers. Table 5 gives the characteristics of each. Table 6 maps them onto the five levels and reports, for each, the number of supporting publications and how many are original studies. Because a source representing more than one unit was assigned to more than one level, the level counts sum to more than 44.

The pattern usually described is that the lower levels are more developed than the higher ones [1,6,12,14]. We do not claim that this gradient is consistent across the literature. Establishing consistency would require comparative counts of externally validated and prospectively evaluated systems by level, reported on a common basis. No source in this corpus provides them, and our own corpus is too small and too unevenly reported to supply them. What can be said is narrower: the reviews we retrieved describe the field in these terms; our own reading of the corpus is partly consistent with them and partly not. Our reading is more qualified, and separating the three dimensions of Table 2 gives a different answer in each. On technical development, physiological and patient twins have the most detailed and best-specified models, and hospital and system work is dominated by architectures. On validation, no level is strong: internal or retrospective evaluation predominates everywhere, external validation is uncommon, and prospective evaluation is rare enough that the finding that only two of 149 studies mentioned verification, validation and uncertainty quantification remains representative, not anomalous [6]. On implementation, nothing in this corpus is reported as being in routine use at any level. Describing the field as mature at the patient level and immature above it therefore conflates a real difference in technical development with an absence of difference in validation and implementation [27,40].

Counting publications is not the same as weighing evidence, and the level counts in Table 6 should not be read as a ranking of evidential strength. Thirteen publications supporting the patient level, of which none reports external validation in an independent population and none reports prospective evaluation, constitute weaker support than a smaller number of externally validated studies would. The corpus is thin at every level on the dimensions that determine strength: whether a finding has been reproduced in a different population, whether it was tested prospectively, whether the comparison was controlled, and whether the system was observed in use. It is thin in a way that quantity conceals [6,17]. Where we describe a level as better represented, we mean that more publications describe applications at it, and nothing stronger.

The first restricted reading reinforces that caution. Original studies here means primary research reports as designated by the publishing journal, including model-development, simulation, architecture-design and formal consensus studies. It does not mean clinical trials, of which the corpus contains none. Restricted to those 10 studies, the distribution differs sharply from the impression given by the corpus as a whole. Seven of them sit at the care-delivery level and one at the physiological level. The standing of physiological and patient twins therefore rests substantially on review and perspective articles, whereas the primary literature we retrieved is weighted towards process and unit modelling. Read from the corpus as a whole, a maturity gradient is in part a gradient in publication type, and the direction of the two gradients is opposite.

The second check could not be completed as intended, and the reason is itself a finding. We set out to restrict the corpus to sources describing systems that satisfy all three definitional criteria of Section 2.3. For most sources this cannot be determined: the update interval and the directionality of coupling are not reported in enough detail to decide whether a described system is a full digital twin, a proto-twin or a shadow. Among the sources that do report these properties, the systems are predominantly personalised and predictive but updated episodically rather than at an interval matched to their decision horizon, and the hospital- and system-level examples are simulations calibrated to aggregate data and not coupled to a named institution. We therefore report an attempted restriction and its failure, not a proportion we cannot defend. That so many sources cannot be classified against the definition they invoke is consistent with the scoping review that found only 18 of 149 publications met equivalent criteria and only two mentioned verification, validation and uncertainty quantification [6]. The practical consequence is that the five-level structure remains usable as a way of sorting what is being represented, whereas any claim about a maturity gradient rests on what sources report about themselves, not on verified system properties.

Several factors may contribute to the observed gradient beyond scale alone. These include differences in the maturity of the underlying research fields, the complexity of implementation across multiple organisations, and the legal and governance requirements associated with sharing data between institutions. Commercially developed systems are not always described in the academic literature. Together, these considerations suggest that organisational and governance complexity, rather than scale itself, may play an important role in shaping the current evidence base [11].

Critical care provides an example in which four of the five levels appear inside a single clinical area. A patient twin represents the individual critically ill patient and supports decisions about that patient’s management, including modelling of clinical trajectories in conditions such as sepsis [47]. A care-delivery twin represents the intensive-care workflow, including patient flow, bed allocation and staffing [31]. A hospital twin represents the institution’s overall critical-care capacity in the context of the rest of its operations [9]. A health-system twin represents the regional response when that capacity is stressed by a crisis, an epidemic or a disaster [33]. The same clinical issue examined across four organisational scales may require distinct but interconnected models. A physiological example, such as a cardiovascular or respiratory-mechanics model personalised to the same patient, would complete the set, and we are not aware of a published example spanning all five levels within one service. Critical care is a reasonable place to test the concept [31,44,49].

An additional question is what happens when digital twins are linked across different levels of the healthcare system. Predictions generated at one level may influence decisions at another, but it remains unclear how uncertainty accumulates as information moves between connected models. We found little discussion of this issue in the healthcare digital twin literature, despite its potential importance for systems that combine models across organisational levels.

Feedback instability is another challenge that deserves greater attention. Once model recommendations begin to influence clinical or organisational practice, they can also change the data used for future model updates. This means that model performance cannot be assumed to remain stable after deployment and highlights the need for continued monitoring and re-evaluation over time [49].

### 3.8. Digital Infrastructure for Multiscale Healthcare Twins

Twins at every scale depend on the availability of data. Physiological and patient twins draw on electronic health records, imaging archives, laboratory information systems, bedside and intensive care monitors, wearable devices and medical equipment [41]. Hospital and system twins add operational sources: hospital management systems, bed-management systems and logistics data. These must be integrated before they can support a twin, which requires shared data infrastructure and interoperable information systems. Current approaches rely on Fast Healthcare Interoperability Resources, data lakes and health data spaces, including the one now being established across the European Union [4,9,12,52,55].

Fast Healthcare Interoperability Resources provides a standard for exchanging healthcare data, but interoperability extends beyond structural exchange. A shared exchange format alone does not resolve challenges related to semantic consistency, governance, or the integration of heterogeneous healthcare systems [52,55]. In our view, infrastructure supporting healthcare digital twins should also consider standardised clinical terminologies, metadata, provenance, lifecycle governance, and mechanisms for maintaining model validity as systems and data evolve.

The location of computation is an important consideration when implementing healthcare digital twins. Time-critical applications often require computation close to the data source, whereas hospital- and health-system-level applications can make greater use of centralised infrastructure. As a result, hybrid architectures that combine local and central computing resources are likely to be common [16]. These choices also affect data governance by influencing where patient data are processed and how responsibility for their management is shared across organisations [11,12].

On top of the data sit the components that make a representation a twin. A modelling layer, whether mechanistic, statistical or a combination of the two, maintains the virtual representation and keeps it aligned with incoming data. A simulation engine runs the candidate scenarios. A governance layer manages access, accountability and audit. The whole system is protected by cybersecurity, since a twin integrates sensitive data and, at the hospital and system scale, becomes part of critical infrastructure [9,11,28]. Comprehensive surveys of human-digital twin architectures describe these layers together with the sensing, connectivity and edge-cloud components required for a functioning digital twin [16].

In this architecture, artificial intelligence is an instrument rather than a defining element, and the two ideas are often treated as one. It may be used to learn predictive models where biological mechanisms are not fully understood, to analyse complex clinical data such as medical images and electronic health records, or to support clinicians by presenting model predictions during decision making [6,8]. However, a risk score produced by a machine-learning model is not, by itself, a digital twin, as discussed in Section 3.1 [6]. Mechanistic models remain essential where biological credibility matters and where data are insufficient to support data-driven learning alone [8].

Artificial intelligence adds another complexity. Evaluating a digital twin is therefore not a one-time exercise. As models evolve, verification, validation and uncertainty quantification need to evolve with them to ensure that predictions remain reliable [17]. Recent work also highlights that predictive accuracy alone is not sufficient for clinical adoption. Long-term monitoring, transparent communication of uncertainty and trustworthiness are equally important for safe clinical use [17,19]. Generative AI is attracting increasing attention and may further expand the capabilities of healthcare digital twins. At the same time, recent literature suggests that progress towards clinical implementation will depend on addressing more fundamental challenges, including consistent definitions, rigorous verification and validation, uncertainty quantification, and establishing safety and trustworthiness [17,36].

## 4. Cross-Cutting Challenges

### 4.1. Adoption and Implementation Across Scales

The challenges associated with digital twins do not belong to any single level. They occur across the healthcare system and change in nature as the focus shifts from individual patients to healthcare infrastructures [11,12]. Technological maturity alone is not enough for implementation [11]. At the patient level, adoption readiness depends mainly on technical and clinical factors. Most applications remain prototypes, and few have been converted from a single-study model into a system used in routine care [8]. Successful implementation in precision medicine depends on computational, practical and regulatory considerations together [28].

As the focus moves from the patient to care delivery and beyond, technical difficulty does not diminish. What happens is that organisational difficulty is added to it [12]. The principal obstacles include integration of heterogeneous information systems, cost, governance requirements, and acceptance by healthcare professionals and institutions. Meta-reviews identify data quality together with ethical and socioeconomic barriers as key obstacles to adoption [12]. Readiness must therefore be assessed separately at each level, and not assumed from the technical maturity of a single model [1,12].

Listing barriers is not the same as describing an implementation process, and this literature does much more of the former. What is largely missing is attention to organisational readiness before adoption, to redesign of the workflow the system enters, to engagement of the staff whose work it changes, to local adaptation of a model developed elsewhere, to what sustains the system once the project funding ends, and to how a system that does not work is withdrawn. Each of these has an established implementation-science vocabulary. Burr et al. explicitly frame the barriers they identify using the non-adoption, abandonment, scale-up, spread and sustainability framework. This perspective is still not common in the broader digital twin literature [11,12]. A recent review of digital twins in disability care states the requirement plainly: integrating such systems into clinical workflows demands changes in service delivery models, staff training and organisational culture. Reimbursement arrangements oriented towards episodic care sit awkwardly with technologies whose value depends on continuous monitoring [43].

Cost is usually named as a barrier without being decomposed, which makes it impossible to act on. The components differ in kind, in timing and in who bears them. Infrastructure is capital expenditure on computing, storage, network and sensing, incurred once and then renewed. Software development is either a build cost or a recurring licence, and the two have different consequences for vendor dependence. Validation is not a single cost but a recurring one, falling due whenever the model, the data model or the population changes. Maintenance covers model updating, revalidation and the engineering effort of keeping a system synchronised with the institution it represents. Cybersecurity is continuous, and not capital. Staffing requires modelling, data-engineering and clinical-informatics skills that most institutions do not currently employ and cannot readily recruit. And there is the opportunity cost of the clinical and managerial time the system consumes, which appears in no budget. Cost-effectiveness against a simpler alternative (a dashboard, a rule, or an extra member of staff) is a further question again, and no source in this corpus answers it.

We should be plain about what this means. We found no cost-effectiveness analysis, no budget-impact assessment and no reported cost per unit of benefit for any healthcare digital twin at any of the five levels. Socioeconomic barriers appear in this literature as a named obstacle, not as a measured quantity [12]. We therefore cannot state what these systems cost, what they save, or at what scale they would repay their construction, and we decline to estimate figures the sources do not contain. What can be said is what an economic evaluation would have to establish before a health system could reasonably invest: the comparator, which is rarely no system at all and is usually a simpler alternative already in place; the perspective, since costs falling on a hospital and benefits accruing to a payer or to patients will not appear in the same account; the time horizon, which must be long enough to include revalidation and maintenance, not construction alone; and the counterfactual, which is the same problem that makes clinical validation difficult in the first place.

The implications extend beyond individual healthcare systems. Digital twins depend on data, digital infrastructure and clinical expertise, resources that are not distributed equally across healthcare settings [12]. This raises an important question about how widely these technologies can be implemented and who is most likely to benefit from them. Digital twins offer real opportunities to improve care, but these benefits cannot be taken for granted. Without careful attention to equity, representation, and accessibility, existing disparities may simply be reproduced in digital form [33,43]. An equally important question is how digital twins can be designed for healthcare settings with more limited infrastructure, for example by relying on intermittently collected data or focusing on unit-level rather than patient-level applications [42,43].

The real challenge begins after deployment, because a digital twin is never finished. As new data are incorporated and the model evolves, its performance has to be reassessed to ensure that predictions remain reliable [17]. But keeping the model up to date is only one part of the equation. Organisations also need the expertise, infrastructure and long-term support to use these systems effectively, and without sustained training, collaboration, governance, and investment, even a well-designed digital twin may never become part of routine clinical care [12]. Continuous operation carries a continuing computational and financial cost, unlike a study that ends when its data are analysed. And deimplementation (deciding that a system is not working, withdrawing it, and reverting to the previous process without harm) is not described anywhere in the sources reviewed here, although it is the outcome for a substantial share of clinical software.

A digital twin is only useful if clinicians are willing to use it. That depends on more than how well the model performs. Usability, automation bias, alert fatigue, and the confidence to question or override AI-generated recommendations all influence how a system is used in practice. Even accurate recommendations may have little value if they do not fit naturally into clinical workflows or support decisions when they are needed most [11,19].

### 4.2. Validation Across Scales: A Staged Pathway

Validation is one of the most persistent weaknesses in healthcare digital-twin research [17]. A useful twin must be accurate enough, and the uncertainty in its predictions understood well enough, to support the decision it is meant to support. This requires verification, validation and uncertainty quantification as separate activities [17]. Formal validation nonetheless remains rare. A scoping review found that only two of 149 studies mentioned verification, validation and uncertainty quantification [6]. Although the exact number depends on the methodology and inclusion criteria used in that review, it still suggests that explicit reporting of verification, validation and uncertainty quantification is uncommon. The studies included in our review showed the same overall pattern.

Validation is used across this literature as a single word covering activities that are not interchangeable. Code verification asks whether the software solves the equations it claims to solve. Model verification asks whether the equations represent the intended system. Calibration fits parameters to observed data. Internal validation estimates performance in the data used for development. External validation tests performance in a different population or site. Temporal validation tests it in a later period. Uncertainty quantification characterises how much confidence the output deserves. Prospective evaluation observes the system running in parallel with real decisions without influencing them. Impact evaluation asks whether decisions and outcomes change when it does influence them. Post-deployment surveillance asks whether it continues to work. These form a staged pathway, and a system that has completed the first four is not validated for clinical use [4,17].

Uncertainty deserves separate treatment, because a twin that reports a single number invites a confidence it has not earned. Several distinct uncertainties combine in any prediction a twin makes. Parameters are estimated from finite and often sparse data, so they carry estimation error. The model structure itself may be wrong, and structural uncertainty is the hardest to quantify because it cannot be estimated from within the model. Measurements entering the model carry error, which propagates. Biological and organisational processes are partly stochastic, so some variation is irreducible however good the model becomes. And where a twin is used to compare interventions, the causal assumptions carry an uncertainty of their own that no confidence interval expresses [4,17].

Quantifying these is one problem; communicating them is another, and it is the one that determines whether a clinician uses the output well. A predicted probability that is well calibrated in aggregate may still be unreliable for an individual patient whose characteristics are sparsely represented in the development data, and a system that presents every prediction with the same visual authority gives the user no way to tell those cases apart. Prediction intervals can be reported instead of point estimates. A system can indicate when an input falls outside the range over which the model was calibrated, and can decline to produce an output where the state estimate is too poorly constrained. All three are available and rarely described. No source in this corpus reports how uncertainty was conveyed to the people expected to act on the output.

Validation is particularly difficult in healthcare because each patient has only one observed clinical course, so model predictions cannot be compared against the outcome that would have followed a different decision. This has been highlighted in work on cancer digital twins [17,18]. Methodological responses exist and are under-used: causal inference with explicitly stated identification assumptions; randomised evaluation of the system, not of the model; synthetic or external control groups where randomisation is not feasible; external validation across sites as a partial substitute for counterfactual comparison; and prospective shadow-mode deployment, in which the system runs and records what it would have recommended while clinicians remain unaware of its output [4,17].

The validation target also changes with scale. Table 7 separates the domains that apply at every level from those specific to a given level, because a single validation question per scale would understate what the lower levels still require and what the higher levels share with them. Calibration, robustness, uncertainty quantification, external validity and safety apply everywhere. What differs is the additional, level-specific question and the outcome against which it is assessed.

Accuracy and utility should not be conflated. A model may predict well and still fail to improve clinical decisions, workflow, safety or patient outcomes, because the decision was already being made correctly, because the prediction arrives too late to act on, or because the action it implies is unavailable [11,17]. Demonstrating impact rather than accuracy is the principal methodological challenge in this field, and it is the stage at which almost all work reviewed here stops [4,17].

### 4.3. Governance, Equity, Security and Regulation

Technical performance alone will not be enough. Healthcare digital twins also require trust, transparency and credible governance [11]. Ethical, legal and social issues become more consequential as a twin connects more data sources and influences a wider population, so protecting sensitive data is a condition of acceptability, and not a secondary design question [9,11]. Healthcare professionals need to understand how a digital twin reaches its recommendations, and responsibility for decisions supported by these systems must be clearly defined [11].

Governance requirements are not uniform across the five levels, and treating them as one set obscures what is actually required. A physiological twin used in research raises questions about data provenance, model transparency and reproducibility. A patient twin raises consent, including the right to withdraw data from a model already trained on it, together with privacy and clinical accountability. A care-delivery twin raises professional responsibility within a team and the ownership of a priority rule the model applies. A hospital twin raises institutional data governance, procurement, vendor lock-in and the approval process for model updates. A health-system twin raises cross-organisational governance, the legal basis for linkage between controllers, public legitimacy and the allocation of public resources [11,28]. Across all levels we would regard an auditable record of what the model recommended and what was decided, a route for reporting incidents, and a named party responsible for adverse decisions as minimum requirements. None of them is described in the sources reviewed here.

Equity is not confined to the system level. Bias can enter at the physiological level through training data that under-represent populations, at the patient level through differential measurement error in devices validated in narrow groups, and at the care-delivery level through unequal access to the technology that generates the data a twin needs [19,42]. Where artificial intelligence forms part of the twin, existing inequalities may be reproduced and amplified unless fairness is treated as a design requirement, not an audit performed afterwards [19,42]. Populations whose care depends on continuous monitoring and adaptive assistive technology are among those with most to gain from continuously updated representations, and are correspondingly exposed if the data from which those representations are built under-represent them [43].

Cybersecurity in a bidirectionally coupled system extends well beyond confidentiality. A system that acts on its counterpart can be attacked through the data it consumes as well as through the outputs it produces: poisoning of training or streaming data, manipulation of the model itself, unauthorised actuation, denial of service at a moment of operational stress, and disruption of infrastructure that a hospital has come to depend on. At institutional and regional scale a twin becomes critical infrastructure, and in our view should be treated as such from the design stage, not after an incident.

Regulation remains unsettled, and the key question is how healthcare digital twins should be classified within existing regulatory frameworks. Digital twins that support the diagnosis, prevention or treatment of individual patients are likely to fall within medical-device and software-as-a-medical-device frameworks. By contrast, twins developed for operational or capacity planning may be subject to different regulatory requirements while still being covered by data protection legislation. The increasing use of machine learning adds another layer of regulatory and governance challenges. At the same time, several features of digital twins make existing frameworks difficult to apply. These systems can evolve continuously as new data become available, they often involve multiple organisations, and they may influence patient care without producing information about a specific patient. Together, these characteristics make questions of validation, responsibility and oversight more difficult to address. As a result, regulatory uncertainty remains a major barrier to implementation and is unlikely to be resolved through technical advances alone [11,28].

Technical development, implementation and regulation therefore cannot be treated separately. Privacy, security, transparency, accountability and fairness need attention during development, not after the technology has been introduced [19,42].

## 5. Discussion

This review followed healthcare digital twins from organs and patients through care processes to hospitals and health systems and organised them along a single axis: what is being represented. Four points follow from the synthesis, and they are interpretive rather than descriptive. The evidence on which they rest is set out in Section 3.

First, the evidence suggests that digital twins are better understood as a multiscale concept than as a single technology. Across all five levels the basic idea remains the same, a twin linked to its physical counterpart through two-way data exchange, but the object being represented ranges from a biological subsystem to a regional network [1,4]. Splitting the literature into physiological, patient, care-delivery, hospital and health-system twins, and reading it as one continuum rather than as separate bodies of literature on cardiac, oncological or hospital twins, is what makes the shared structure visible [14]. The proposed framework is intended as an analytical framework rather than an established classification. Its relationship to existing classification schemes is summarised in Table 4.

Second, the evidence differs across levels, but not in the way the field usually describes. Physiological and patient twins are more developed technically, with concrete mechanistic and clinical examples drawn most strongly from cardiology and oncology [30,35,37]. Hospital and health-system twins are still represented mainly by conceptual frameworks, proposed architectures and early applications rather than validated systems [1,9]. On validation and implementation, however, the levels are more alike than different: external validation is uncommon everywhere, prospective evaluation is rare everywhere, and routine deployment is absent everywhere in this corpus [6,17]. Calling the patient level mature overstates what the evidence supports; relatively more developed is the accurate description [27,40].

Third, the main challenge also changes with scale. For patient twins the binding constraint is usually developing an accurate, appropriately updated model from individual data [8]. For hospital and system twins that constraint persists, since semantic harmonisation, distributed computation, latency, data quality and model coupling are all unresolved, while organisational, policy and distributive questions are added on top [11,12]. It would be misleading to say that the obstacle moves away from technical accuracy. The obstacles accumulate; they do not substitute for one another. Describing the higher-level difficulties as political is also too coarse. They separate into policy choice, distributive justice, institutional accountability, public legitimacy and cross-organisational governance, which have different remedies and different responsible parties [11].

Fourth, health system intelligence as defined in Section 1 would require twins that are validated, connected and governed together, and none of the three is currently in place. Without validation the outputs cannot be relied upon, without interoperability the levels cannot be connected, and without governance the system cannot be used on real populations. The relative technical development of physiological and patient twins suggests, as a hypothesis rather than a conclusion, that the earliest clinical benefits may appear at those levels, particularly in treatment planning and prognosis. This review did not compare clinical-effectiveness evidence systematically and cannot establish it. Higher-level twins would require different conditions again, in which heterogeneous data integration, interoperability, institutional trust and organisational design matter as much as modelling, and in which data protection, security, explainability, accountability and bias mitigation are design requirements, and not later additions [11,12,19,42].

Different definitions should be considered carefully rather than assuming that one is the only correct one. Frameworks that require continuous coupling and those that accept periodic or human-mediated updating lead to different interpretations of the same field. Under the stricter definition, many published systems would no longer qualify as digital twins. Under the broader definition, the concept becomes so broad that it loses its ability to distinguish between different types of systems [6,26]. Boundary cases help illustrate where the disagreement lies. Examples include a cardiac model personalised from a single imaging study and then run forward, an epidemic simulation calibrated to national surveillance data, and a hospital model designed but never connected to a live data feed. Each is classified differently under different frameworks, and each appears in this corpus. We have applied the stricter definition and reported what it excludes, which is a position rather than a finding.

The findings have different implications for different audiences. For researchers, an important priority is to move beyond developing new architectures and towards external and prospective validation of systems that already exist, together with clearly stating the causal assumptions on which a model is expected to support intervention [6,11,12]. For clinicians, the key questions are what a model’s output means, what its limitations are, and where responsibility lies when its recommendations are followed or overridden [11]. For hospital managers, deciding whether to invest in digital twins remains challenging because their value is still uncertain. In practice, these decisions are likely to be more effective when driven by clearly defined operational needs rather than by the technology itself [11,12]. For policymakers and regulators, important priorities include determining how these systems fit within existing medical device and artificial intelligence frameworks, establishing appropriate legal mechanisms for cross-organisational data sharing, and clarifying accountability when model-informed decisions lead to adverse outcomes [11,19].

Compared with earlier reviews, this article focuses on the relations between levels. Scoping reviews have mapped applications and terminology, meta-reviews have grouped use cases and implementation barriers, and bibliometric studies have documented the growth of the field [1,6,12]. What they leave less developed is the movement between the organ, patient, care process, hospital and system, which reviews organised by disease, organ or technology tend to keep apart [14,26,27,40]. The contribution of this review is to propose and examine these levels as a connected framework and to assess how the concept changes as it moves from the individual patient to collective settings.

### 5.1. Practical Applicability of the Proposed Framework

The proposed framework is intended as a practical tool as well as an analytical one. By distinguishing digital twins according to the object they represent, it provides a consistent way to classify applications that might otherwise be grouped only by clinical specialty or technology. This can help researchers compare studies more consistently and support developers and decision-makers in selecting the appropriate level for a digital twin application. The framework may also help guide evaluation and implementation. Because each level is associated with different validation questions, it can help identify the next stage of development for a particular application. It also highlights that governance and accountability differ across levels, meaning that the requirements for a patient-level digital twin are not necessarily the same as those for a hospital or health-system digital twin.

### 5.2. Future Directions

Recommendations for improved validation and governance are more useful when linked to specific research priorities. Prospective multi-centre external validation of existing patient-level twins in populations other than the development population would establish transportability, which is currently assumed rather than demonstrated. Temporal validation across a period of practice change would establish stability. Shadow-mode deployment would show how often system recommendations differ from routine clinical decisions. Randomised evaluation of the system, comparing units or periods with and without access to its output, would establish its real-world impact. None has been reported in this corpus [4,17]. Human-factors studies of override behaviour and alert response would show whether output is used as intended. Economic evaluation alongside any operational deployment would address the cost question that the hospital-level literature leaves open. Post-deployment surveillance protocols would help detect model drift and determine when revalidation is required [17,19].

Beyond validation, the integration of artificial intelligence with the mechanistic models that remain central to twins is an open methodological question, and recent work has begun to explore it [36]. Foundational research agendas identify this, together with the data infrastructures twins require, as priority areas [4]. In our view, the field would benefit from clearer and more consistent definitions. At present, variation in how the term digital twin is used makes it more difficult to compare studies and interpret the evidence consistently [6].

Implementation challenges are discussed in Section 4.1. Four seem particularly important. First, digital twins require access to live operational data and cannot be evaluated using static datasets alone. Second, maintaining and updating models over time remains an important organisational challenge [11,12]. Third, successful implementation depends on integrating the system into existing clinical workflows, together with staff engagement and training [12,43]. Finally, the reviewed literature provides little guidance on how systems should be withdrawn when they no longer perform as intended.

### 5.3. Limitations

The search, study selection and classification were carried out by a single author without independent duplicate screening, inter-rater agreement or a formal consensus process. We also did not perform a formal quality appraisal or risk-of-bias assessment. Together, these methodological limitations should be considered when interpreting the maturity assessments presented in this review, particularly in a field where the term “digital twin” is used inconsistently [6]. The five-level framework was developed during the review and reflects our interpretation of the literature rather than the results of a formal qualitative analysis. Finally, Table 5 summarises the characteristics that could be reported consistently across the included studies, but a detailed study-level evidence table was beyond the scope of this narrative review.

Because the evidence base includes both review articles and some of the primary studies they summarise, certain findings may have influenced the overall conclusions more than once, although primary studies identified only through reviews were not added separately. Citation bias is also possible, as highly cited reviews are more likely to be retrieved. Publication bias is another limitation, since unsuccessful prototypes and abandoned implementations are rarely reported, meaning that the published literature is more likely to reflect successful applications. Excluding grey literature may also have omitted operational work undertaken by healthcare organisations and commercial developers. In addition, the date restriction was a pragmatic decision, maturity assessments remained subjective, and confirmation bias cannot be excluded because the framework was developed and applied by the same authors. We sought to reduce this risk by presenting alternative interpretations in Section 3.7 and by acknowledging where the framework did not fit particular applications.

The available evidence is also geographically and clinically uneven. Where study setting was reported, most studies originated from high-income healthcare systems, which may limit the transferability of the findings to settings with different infrastructure, regulatory environments, workforce capacity or funding models. Clinically, the literature is concentrated in cardiology, oncology, critical care and other technology-intensive settings, whereas primary care, mental health, maternity care and long-term care are represented much less frequently. These findings should therefore be interpreted cautiously in those settings.

Finally, the proposed five-level framework is intended as an analytical framework rather than an established classification. Restricting the review to English-language publications may also have excluded relevant studies, and the field continues to evolve rapidly.

## 6. Conclusions

Healthcare digital twins are no longer limited to the individual patient. Their applications extend to care processes, hospitals and health systems, and viewing these levels as a continuum is what turns a scattered set of applications into a single framework. That five-level framework is proposed here as an analytical framework rather than offered as an established classification.

Physiological and patient twins are more frequently represented by concrete applications in the reviewed literature. Although it is reasonable to hypothesise that the first clinical benefits may emerge in these areas, this review did not systematically compare clinical-effectiveness evidence and therefore cannot support a stronger conclusion. Hospital and health-system twins remain largely at the research stage [1,9].

As healthcare digital twins move from patients to hospitals and health systems, each level introduces additional requirements while retaining the technical challenges of the previous one. Interoperability, organisational and governance requirements also become increasingly prominent.

Health system intelligence, in the sense defined in Section 1, would likely require digital twins across scales to be validated, connected and governed together. Prospective evidence of patient benefit, operational effectiveness and system-level impact remains limited at every level of the framework. Strengthening this evidence base should therefore be a priority for future research [6,17].

## Figures and Tables

**Table 1:** Search strategy by source, showing platform, complete search string and filters applied.

Source	Platform	Search String as Run	Filters Applied	Date Run
Web of Science Core Collection	Clarivate	TS=(“digital twin*”) AND TS=(patient* OR “precision medicine” OR “care delivery” OR hospital* OR “health system*” OR “public health” OR “intensive care” OR workflow* OR healthcare OR “health care”)	Document types: article, review. Language: English. Timespan 2022–2026.	June–July 2026
Scopus	Elsevier	TITLE-ABS-KEY(“digital twin*”) AND TITLE-ABS-KEY(patient* OR “precision medicine” OR “care delivery” OR hospital* OR “health system*” OR “public health” OR “intensive care” OR workflow* OR healthcare OR “health care”) AND PUBYEAR > 2021	LIMIT-TO(DOCTYPE, “ar”, “re”); LIMIT-TO(LANGUAGE, “English”).	June–July 2026
PubMed	NCBI	(“digital twin”[Title/Abstract] OR “digital twins”[Title/Abstract] OR “digital twinning”[Title/Abstract]) AND (“Precision Medicine”[MeSH] OR “Delivery of Health Care”[MeSH] OR “Hospitals”[MeSH] OR “Intensive Care Units”[MeSH] OR “Public Health”[MeSH] OR “Workflow”[MeSH] OR patient*[Title/Abstract] OR “precision medicine”[Title/Abstract] OR “care delivery”[Title/Abstract] OR hospital*[Title/Abstract] OR “health system*”[Title/Abstract] OR “intensive care”[Title/Abstract] OR workflow*[Title/Abstract])	Publication date 2022/01/01 to date last run. Language: English.	June–July 2026
Google Scholar	Google	“digital twin” AND (healthcare OR hospital OR “health system” OR “precision medicine”); used only as a supplementary check for records missed by the indexed databases.	Sorted by relevance; custom range 2022–2026. The number of ranked records screened before stopping was not recorded, so this source is reported for completeness and is not presented as reproducible.	June–July 2026

**Table 2:** Labels used to record the definitional status of each described system and its position on three separate dimensions of maturity. Labels were assigned by the authors from what each source reported; no critical-appraisal instrument was applied.

Dimension	Label	Definition Applied in This Review
Definitional status	Full digital twin	Represents an identified counterpart, is updated from data generated by that counterpart at an interval matched to the decision horizon and produces decision-relevant predictions.
	Proto-twin	Personalised to an identified counterpart and predictive, but updated only at initialisation or episodically, so synchronisation is not matched to the decision horizon.
	Digital shadow	Updated from its counterpart, possibly continuously, with no coupling running back from model output to the counterpart.
	Dynamic simulation	Time-evolving model calibrated to aggregate or population data rather than to an identified counterpart.
	Predictive model	Produces a prediction or risk estimate without a maintained representation of a counterpart.
	Conceptual architecture	Specifies components, data flows or requirements without an implemented system.
Technical development	Conceptual proposal	Design or requirements described; no working implementation reported.
	Prototype	Implemented and run on retrospective or synthetic data, or in a single limited setting.
	Operational system	Runs continuously against live data from its counterpart.
Validation	None reported	No verification, calibration or performance assessment described.
	Internal or retrospective	Performance assessed in the development data or in a retrospective sample from the same source.
	External or temporal	Performance assessed in a different site or population, or in a later time period.
	Prospective observational	System run in parallel with real decisions without influencing them (shadow mode).
	Comparative impact	Decisions or outcomes compared with and without the system, randomised or otherwise controlled.
Implementation	None	No use outside the study environment.
	Single-site pilot	Used within one service for a defined period, usually alongside existing practice.
	Routine use	Embedded in the standard workflow of one or more services beyond a pilot period.

**Table 3:** The five proposed levels, with the physical counterpart, principal data sources, dominant modelling approach, decisions supported, separated maturity dimensions and the principal validation and governance challenge at each level. Maturity entries are provisional author judgements assigned using the labels in Table 2; no critical-appraisal instrument was applied.

Level of Digital Twin	Physical Counterpart	Main Data Sources and Modelling Approach	Decisions Supported	Typical Time Scale and Decision-Maker	Maturity: Technical/Validation/Implementation	Principal Validation and Governance Challenge
Physiological twin	Organ, tissue or biological subsystem	Molecular, cellular, imaging and physiological data; mechanistic multiscale models	Understanding biological mechanisms	Seconds to months; researcher, and the clinician interpreting the output	Prototype/internal, some external/none	Validation: mechanistic adequacy and parameter identifiability, not biological plausibility alone. Governance: data provenance and model transparency.
Patient twin	The individual patient	Electronic health record, imaging, genomics, wearables and device data; combined mechanistic and statistical models	Personalised care and prognosis	Hours to years; clinician together with the patient	Prototype/mostly internal/none	Validation: predictive performance in an individual patient, and transportability between populations. Governance: privacy, consent and withdrawal, clinical accountability.
Care-delivery twin	A clinical process, pathway or unit	Clinical, workflow, staffing and patient-flow data; discrete-event and process simulation, sometimes combined with predictive models	Improving workflow, timing and resource use	Minutes to days; clinical team and unit lead	Prototype/internal/single-site pilot at most	Validation: demonstrated improvement in workflow, safety, timing and resource use. Governance: professional responsibility within the unit, and ownership of the priority rule the model applies.
Hospital twin	The institution as an operating system	Clinical, operational and hospital management data; operational simulation with integrated data	Capacity, staffing, logistics and reorganisation	Hours to seasons; hospital management	Conceptual proposal to prototype/little reported/none	Validation: operational efficiency, occupancy, staff allocation and resilience. Governance: institutional data governance, interoperability, procurement and model-update approval.
Health-system twin	A regional or population health system	Multi-organisation, population and public-health data; system- and population-level simulation	Capacity planning, preparedness and resource allocation	Weeks to years; commissioners and public authorities	Largely conceptual/little reported/none	Validation: equity, access, preparedness and cost-effectiveness, none of which is currently operationalised. Governance: cross-organisation governance, public resources, legitimacy and trust.

**Table 4:** The proposed five-level framework compared with existing classifications of healthcare digital twins, showing the organising axis of each, what it establishes, and what it leaves unresolved for a cross-scale reading.

Classification	Organising Axis	What It Establishes	What It Leaves Unresolved for a Cross-Scale Reading
Scoping review of digital twins for health [1]	Clinical domain and data type	Breadth of application areas and the data modalities in use	Levels are not distinguished, so a cardiac model and a hospital architecture appear in the same inventory
Scoping review of human digital twins [6]	Compliance with a definition	How rarely published systems meet a full digital-twin definition	Applies one threshold across the field without asking whether the threshold should differ by represented unit
Meta-review of applications and implementation challenges [12]	Purpose of the application	Three application groups (personalised medicine, operational efficiency, research) and the barriers common to them	Purpose and scale cross-cut: operational efficiency spans unit, hospital and region, which have different validation and governance requirements
Bibliometric literature review [14]	Publication pattern	Growth, geography and disciplinary spread of the field	Describes the literature rather than the objects it studies
Scoping review of clinical and operational decision-making [34]	Type of decision supported	A clear separation between clinical and operational uses	A binary split places unit, institution and region together despite different decision-makers and accountability
Survey of human digital-twin architectures [16]	Technical architecture layer	Sensing, connectivity, edge–cloud and modelling components required for a working system	Architecture is largely scale-invariant, so it does not discriminate between what is being represented
Framework proposed in this review	Unit of representation	A single axis along which validation questions, decision horizons, decision-makers and governance requirements can be compared	Does not resolve boundary cases at the physiological–patient and care-delivery–hospital margins, which are handled by explicit rule and multi-level assignment; and is an analytical proposal, not an empirically derived classification

**Table 5:** Characteristics of the 44 publications forming the evidence base: publication type, clinical or operational domain, the level or levels to which each was assigned, and, for the original studies, the country of the study setting, the validation design and the deployment status; these last three are not applicable to reviews and conceptual sources, which report no system of their own. Sources representing more than one unit are assigned to more than one level, so level counts in Table 6 exceed the number of publications. The remaining fields requiring full-text extraction of system properties are discussed in Section 2.5 and are not tabulated.

Ref.	Source	Country	Publication Type	Clinical or Operational Domain	Validation Design	Deployment Status	Level or Levels Assigned
[1]	Katsoulakis et al. (2024)	Not applicable	Review or evidence synthesis	Healthcare, cross-domain	Not applicable	Not applicable	Hospital; cross-cutting
[2]	Li X. et al. (2025)	Not applicable	Conceptual, perspective or consensus	Preventive and personalised medicine	Not applicable	Not applicable	Health-system
[3]	Vallée (2023)	Not applicable	Conceptual, perspective or consensus	Health systems and hospital operations	Not applicable	Not applicable	Hospital; health-system
[4]	National Academies (2024)	Not applicable	Conceptual, perspective or consensus	Cross-sector research agenda	Not applicable	Not applicable	Cross-cutting
[6]	Tudor et al. (2025)	Not applicable	Review or evidence synthesis	Human digital twins, cross-domain	Not applicable	Not applicable	Cross-cutting
[7]	Venkatesh et al. (2024)	Not applicable	Review or evidence synthesis	Life science and healthcare innovation	Not applicable	Not applicable	Patient
[8]	Laubenbacher et al. (2024)	Not applicable	Conceptual, perspective or consensus	Medicine, general	Not applicable	Not applicable	Patient
[9]	Noeikham et al. (2024)	Thailand	Original study	Healthcare unit operations	Single case study at an outpatient chemotherapy centre; no quantitative performance evaluation reported	Proposed architecture; not deployed	Care-delivery; hospital
[10]	Shen et al. (2024)	Not applicable	Review or evidence synthesis	Population precision health	Not applicable	Not applicable	Health-system
[12]	Ringeval et al. (2025)	Not applicable	Review or evidence synthesis	Cross-domain; implementation	Not applicable	Not applicable	Care-delivery; hospital; cross-cutting
[14]	Machado & Berssaneti (2023)	Not applicable	Review or evidence synthesis	Healthcare, general (bibliometric)	Not applicable	Not applicable	Cross-cutting
[11]	Burr et al. (2026)	Not applicable	Review or evidence synthesis	Ethical, legal and social issues	Not applicable	Not applicable	Cross-cutting
[24]	Sun et al. (2022)	Not applicable	Conceptual, perspective or consensus	Medicine, general	Not applicable	Not applicable	Cross-cutting
[28]	Venkatesh et al. (2022)	Not applicable	Conceptual, perspective or consensus	Precision medicine; regulation	Not applicable	Not applicable	Patient
[29]	Li X. et al. (2022)	Sweden (validation samples also from Austria)	Original study	Molecular and single-cell; drug targets	In vitro experimental validation: time-series scRNA-seq from 16 patients and 14 controls, with an independent antibody-blocking validation set	Research framework; not deployed	Physiological
[30]	Sel et al. (2024)	Not applicable	Review or evidence synthesis	Cardiovascular	Not applicable	Not applicable	Physiological
[31]	Zhong et al. (2022)	United States	Original study	Critical care delivery	Qualitative process mapping coupled with quantitative simulation, built on intensive care data from one academic centre	Research model; not deployed	Care-delivery
[35]	Coorey et al. (2022)	Not applicable	Review or evidence synthesis	Cardiovascular	Not applicable	Not applicable	Physiological
[36]	Thangaraj et al. (2024)	Not applicable	Review or evidence synthesis	Cardiovascular; generative methods	Not applicable	Not applicable	Physiological
[37]	Wu et al. (2022)	Not applicable	Review or evidence synthesis	Clinical oncology	Not applicable	Not applicable	Physiological
[38]	Wang et al. (2024)	Not applicable	Review or evidence synthesis	Immuno-oncology	Not applicable	Not applicable	Physiological
[39]	Ștefăniga et al. (2024)	Not applicable	Review or evidence synthesis	Precision oncology	Not applicable	Not applicable	Physiological
[26]	Zhang et al. (2024)	Not applicable	Review or evidence synthesis	Healthcare and medicine, general	Not applicable	Not applicable	Patient
[40]	Sun et al. (2023)	Not applicable	Review or evidence synthesis	Healthcare, general	Not applicable	Not applicable	Patient
[27]	Armeni et al. (2022)	Not applicable	Review or evidence synthesis	Evidence-based medicine	Not applicable	Not applicable	Patient
[18]	Stahlberg et al. (2022)	Not applicable	Conceptual, perspective or consensus	Oncology	Not applicable	Not applicable	Patient
[44]	Halpern et al. (2025)	Not applicable	Review or evidence synthesis	Critical and acute care	Not applicable	Not applicable	Care-delivery
[45]	Cannon et al. (2024)	United States	Original study	Haemorrhagic shock resuscitation	Mechanistic differential-equation model fitted to porcine experimental data and validated against the human PROMMTT trauma cohort	Research model; model code publicly available	Patient; care-delivery
[46]	Dang et al. (2023)	United States	Original study	Acute stroke, neurocritical care	DELPHI expert consensus, 18 neurocritical care experts, consensus threshold 80%; no patient-data validation	Rule set for a model in development; not deployed	Care-delivery
[47]	Hou et al. (2025)	United States	Original study	Sepsis, intensive care	Retrospective cohort of 19,177 sepsis patients over an 8-year period; unsupervised two-stage clustering; no external validation reported	Analysis informing twin development; not deployed	Patient; care-delivery
[48]	Li H. et al. (2025)	Not applicable	Conceptual, perspective or consensus	Emergency care	Not applicable	Not applicable	Care-delivery
[50]	Xue et al. (2025)	China	Original study	Elective surgical scheduling	Numerical experiments benchmarking the proposed scheduling algorithm; simulation-based comparison	Simulation prototype; not deployed	Care-delivery; hospital
[51]	Aluvalu et al. (2023)	India	Original study	Emergency hospital services	Proposed model; no empirical validation reported	Proposed model; not deployed	Hospital
[34]	Riahi et al. (2025)	Not applicable	Review or evidence synthesis	Clinical and operational decision-making	Not applicable	Not applicable	Care-delivery; hospital
[49]	Penverne et al. (2024)	France	Original study	Emergency call response, regional	Discrete-event simulation of four emergency call centres, built on real call data from one peak week; counterfactual scenario comparison	Simulation used for organisational assessment; not deployed	Care-delivery; health-system
[33]	Græsbøll et al. (2024)	Denmark	Original study	COVID-19 testing and lockdown policy	Individual-based simulation replicating the Danish population, calibrated to national surveillance data; counterfactual scenario analysis	Retrospective policy evaluation; model code publicly released	Health-system
[32]	Khan et al. (2022)	Not applicable	Review or evidence synthesis	COVID-19 pandemic response	Not applicable	Not applicable	Health-system
[53]	Elkefi & Asan (2022)	Not applicable	Review or evidence synthesis	Health system management	Not applicable	Not applicable	Health-system
[15]	El-Warrak & Miceli de Farias (2025)	Not applicable	Review or evidence synthesis	Public health	Not applicable	Not applicable	Health-system
[54]	Vallée (2024)	Not applicable	Review or evidence synthesis	Personalised medicine	Not applicable	Not applicable	Patient; health-system
[41]	Johnson & Saikia (2024)	Not applicable	Review or evidence synthesis	Wearable devices	Not applicable	Not applicable	Patient
[16]	Chen et al. (2024)	Not applicable	Review or evidence synthesis	Networking architecture for human twins	Not applicable	Not applicable	Patient
[17]	Sel et al. (2025)	Not applicable	Review or evidence synthesis	Verification, validation and uncertainty	Not applicable	Not applicable	Cross-cutting
[43]	Olawade et al. (2026)	Not applicable	Review or evidence synthesis	Disability care	Not applicable	Not applicable	Patient

**Table 6:** Evidence map: publications supporting each level, by publication type, with the provisional maturity judgement on each of the three dimensions defined in Table 2. Sources representing more than one unit appear at more than one level.

Level	Supporting Publications	n	Original Studies	Technical Development	Validation	Implementation
Physiological twin	[29,30,35,36,37,38,39]	7	1	Prototype	Internal; some external	None reported
Patient twin	[7,8,16,18,26,27,28,40,41,43,45,47,54]	13	2	Prototype	Mostly internal	None reported
Care-delivery twin	[9,12,31,34,44,45,46,47,48,49,50]	11	7	Prototype	Internal	Single-site pilot at most
Hospital twin	[1,3,9,12,34,50,51]	7	3	Conceptual proposal to prototype	Little reported	None reported
Health-system twin	[2,3,10,15,32,33,49,53,54]	9	2	Largely conceptual	Little reported	None reported
Cross-cutting or multilevel	[1,4,6,11,12,14,17,24]	8	0	Not applicable	Not applicable	Not applicable

**Table 7:** Validation domains by level, separating those required at every level from the additional level-specific question, and mapping each to the stage of the pathway at which it is assessed. Legitimacy and public acceptability are listed separately from validation because they are not predictive or operational properties. The level-specific questions are the authors’ formulation, whereas the final column summarises the maturity reflected in the reviewed literature.

Level	Domains Required at Every Level	Additional Level-Specific Question	Stage of the Pathway at Which It Is Currently Assessed
Physiological twin	Code and model verification; calibration; internal and external validation; uncertainty quantification; robustness to missing or degraded input; safety of any action informed	Is the mechanism represented adequately, and are its parameters identifiable from the data available?	Verification and internal validation; external validation in a minority of studies
Patient twin	As above	Does the model predict accurately for this individual, and does that performance transfer to another population?	Internal and retrospective validation; prospective evaluation rare
Care-delivery twin	As above	Does use of the model change workflow, timing, safety or resource use, and does it do so without displacing attention from elsewhere?	Simulation-based evaluation; comparative impact evaluation not reported in this corpus
Hospital twin	As above	Does the model remain synchronised with a live institution, and does its use improve occupancy, staff allocation and resilience without degrading safety?	Architecture description and prototype demonstration
Health-system twin	As above	Does the model support allocation decisions that are accurate, and how are equity, access, preparedness and cost-effectiveness weighed against one another?	Scenario simulation; no reported evaluation against realised system outcomes
All levels: legitimacy and acceptability	Not a validation domain	Do patients, professionals and the public accept the system, and is there an auditable record and a named party accountable for decisions it informs?	Addressed conceptually in the ethical and governance literature; not evaluated empirically in this corpus

## Data Availability

No new primary data were generated. The materials produced during this review are reported in the article itself: the complete database-specific search strings (Table 1), the labelling scheme applied to every included source (Table 2), the characteristics and level assignment of each of the 44 included publications (Table 5), and the level-by-level evidence map (Table 6). Record-level screening yields were not retained during the conduct of the review and therefore cannot be deposited.

## Abbreviations

The following abbreviations are used in this manuscript:

AI	artificial intelligence
COVID-19	coronavirus disease 2019
EHR	electronic health record
FHIR	Fast Healthcare Interoperability Resources
ICU	intensive care unit
PRISMA	Preferred Reporting Items for Systematic Reviews and Meta-Analyses
3D	three-dimensional
